# Chemical and sensory effects of macro-oxygenation and air sparging coupled with oxidation-reduction potential (ORP) monitoring in Syrah wines^☆^

**DOI:** 10.1016/j.fochx.2025.103135

**Published:** 2025-10-04

**Authors:** William Jordan Wright, Sean Kuster, Biljana Petrova, Jesus Villalobos, James Nelson, Robert Coleman, Luis Federico Casassa

**Affiliations:** a Wine and Viticulture Department, California Polytechnic State University San Luis Obispo (Cal Poly), San Luis Obispo, California 93407, USA; b Department of Electrical and Computer Engineering, University of California Davis (UCD), Davis, California 95616, USA; c MeshVines, Sacramento, California 95814, USA; d Department of Viticulture and Enology, Washington State University, Richland, Washington 99354, USA

**Keywords:** Air sparging, aromatics, macro‑oxygenation, redox potential, oxygen, phenolic, punch down

## Abstract

The chemical and sensory effects of different cap management regimes and ORP modulation during red wine alcoholic fermentation were investigated in Syrah wines produced with punch downs (PD); air sparging triggered at oxidation-reduction potential (ORP) –40 mV (RedoxCon); macro‑oxygenation with oxygen (MOX); and double-rate macro‑oxygenation with oxygen (2MOX). RedoxCon received 1125 % and 513 % more O_2_/L than MOX and 2MOX. ORP values were lowest in MOX and 2MOX, leading to wines with 30 % and 11 % more color, 41 % and 27 % more tannin, and 31 % and 32 % more total phenolics relative to PD and RedoxCon, respectively. MOX and 2MOX contained 61 % and 53 % more esters than PD, whereas terpenoids were higher in PD and RedoxCon. RedoxCon enhanced spicy and floral aromas, 2MOX intensified fruit aromas; both MOX and 2MOX increased reduction aroma. Macro‑oxygenation enhanced phenolics and preserved esters by maintaining lower redox environments than the other treatments and possibly lessening volatile stripping. Cool climate Syrah can accommodate oxygen additions over an 11-fold range during alcoholic fermentation, resulting in wines with distinct chemical and sensory features.

## Highlights

• Macro-oxygenation boosts the phenolic profile of Syrah wines.
• Macro-oxygenation preserves sulfur dioxide and glutathione in Syrah wines.
• Redox-controlled air sparging (-40 mV) lowered perceived reduction in Syrah wines.
• Redox-controlled air sparging (−40 mV) increased floral aroma in Syrah wines.
• Syrah wines can accommodate a 11-fold range of O_2_ add during ferment.

## Graphical abstract

**Figure:**
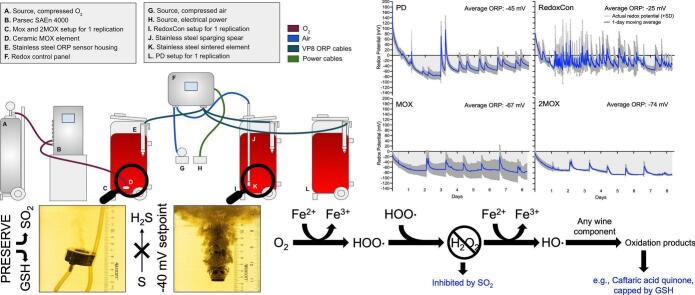

## 1 Introduction

Syrah is a well-established wine grape variety that originated in Southeastern France and is now widely cultivated across both the Old and New Worlds, with over 1531 tons crushed in California in 2023, accounting for 4.1 % of the state's total red wine production (*California Department of Food and Agriculture. (2024, March 8). California grape crush,* 2024). Cool climate Syrah grapes typically show high concentrations of anthocyanins, seed-derived monomers (including monomeric flavan-3-ols such as (+)-catechin, (−)-epicatechin, (−)-epigallocatechin, and (−)-epicatechin-3-*O*-gallate), and volatiles associated with the perception of blackberry, mint, and black pepper aromas (Gil-Muñoz et al., 2009; Parker et al., 2007). Although inherently rich in phenolic and aromatic constituents, the final chemical and sensory profiles of Syrah wines are shaped by winemaking techniques applied during alcoholic fermentation. Studies have shown that cap management protocols such as punch downs (Frost et al., 2018), pump overs (Fischer et al., 2000), submerged cap, extended maceration (Stoffel et al., 2025), and gas mixing (Parnigoni et al., 2025) exert a profound influence on the phenolic and aromatic composition of red wines. To fully understand the effects of these techniques, the main biochemical events that occur during alcoholic fermentation must be considered.

During alcoholic fermentation, *Saccharomyces cerevisiae* metabolizes fermentable sugars, producing carbon dioxide (CO_2_) as a byproduct. Generated CO_2_ causes grape solids to rise, forming a cap. Simultaneously, CO_2_ displaces oxygen (O_2_) and strips volatile compounds from the must (Cerda-Drago et al., 2016; Mouret et al., 2012). Since O_2_ is essential for yeast viability, its reintroduction during alcoholic fermentation is critical. However, measuring dissolved O_2_ in the hypoxic environment of an alcoholic fermentation is challenging due to low native O_2_ concentrations, fast consumption by yeast, and sensor limitations (Kjaergaard, 2006). Although more recent studies have shown the efficacy of improved dissolved O_2_ measurement technology (Picariello et al., 2020; Zhang et al., 2024), the measured values provide only a surface-level view of the impact of oxygenation in must undergoing alcoholic fermentation.

While not a direct measurement of dissolved O_2_, oxidation-reduction potential (ORP) provides insight into the oxidative environment of fermenting must and the outcomes of alcoholic fermentations (Nelson et al., 2025). ORP is measured in millivolts (mV) and reflects the net outcome of oxidation-reduction reactions in a solution (Kjaergaard, 2006). During alcoholic fermentation, when ORP is lowest, the ORP trajectory of the ferment likely reflects the production of ethanol, glycerol, and glutathione (reduced compounds) by *S. cerevisiae* and therefore yeast metabolism (Berovic, 2024; Nelson et al., 2022). After the completion of alcoholic fermentation, different redox couples begin to dominate the ORP. O_2_ cannot directly oxidize organic compounds in its normal triplet form (Danilewicz, 2003). However, O_2_ can accept electrons from reduced metal ions such as Fe^2+^ (ferrous ion) and Cu^+^ (cuprous ion), which oxidize to Fe^3+^ (ferric ion) and Cu^2+^ (cupric ion). The acceptance of an electron by O_2_ likely increases the ORP and produces reactive oxygen species (ROS), particularly HOO• (hydroperoxyl radical) and hydrogen peroxide, H_2_O_2_ (Elias & Waterhouse, 2010). H_2_O_2_ proceeds to participate in the Fenton reaction, oxidizing Fe^2+^ to Fe^3+^ and producing HO• (hydroxyl radical). HO•, a powerful oxidizing agent, oxidizes whichever compound it encounters proportionally to its concentration (Elias & Waterhouse, 2010).

Sulfur dioxide (SO_2_) is widely used in winemaking to control oxidation. SO_2_ in the form of bisulfite prevents chemical oxidation by reducing quinones back to phenols (Nikolantonaki & Waterhouse, 2012) and reducing H_2_O_2_ (formed by the terminal reaction of two HOO•) to H_2_O (water). In doing so, bisulfite is oxidized to sulfate (Danilewicz, 2016). Bisulfite also masks the sensory perception of oxidation in wine by binding aldehydes (Miao & Waterhouse, 2025). The two former mechanisms likely drive ORP down by preventing H_2_O_2_ from participating in the Fenton reaction and quinones from oxidizing other polyphenols (Oliveira et al., 2016). Furthermore, additions of about 50 mg/L SO_2_ at crush to grape must have been shown to minimize polyphenol oxidase (PPO) activity to 2 units/mL, with 100 mg/L SO_2_ completely inhibiting PPO activity (Wissemann & Lee, 1980). In addition to SO_2_, naturally occurring (and added) antioxidants such as glutathione and ascorbic acid play key roles in limiting oxidation. Reduced glutathione (GSH), a tripeptide found in grape skins, swiftly reacts with quinones (chiefly, with the quinone of caftaric acid) to form the grape reaction product (GRP), which cannot be further oxidized (Singleton et al., 1985; Singleton & Cilliers, 1995). GRP limits the pool of oxidizable phenols and likely helps maintain lower ORP values. Similarly, ascorbic acid reduces quinones back to their phenolic forms, likely driving ORP down (Danilewicz, 2003). These powerful nucleophiles, although they can be added, are naturally concentrated in grape solids, making their extraction highly dependent on cap management strategies during alcoholic fermentation (Singha & Das, 2014).

Punch down, a traditional technique, physically and temporarily submerges the cap by plunging. The plunging action mechanically ruptures phenolic-containing vacuoles within the cells of red grape skins and seeds, thereby facilitating the release of anthocyanins and tannins, which subsequently diffuse into the fermenting must (Morata, 2019). Although punch downs promote phenolic extraction, they require labor and introduce unquantifiable amounts of O_2_. They may also preferentially increase the extraction of flavan-3-ols, which are largely confined to seeds and associated with the perception of bitterness (Frost et al., 2018; Parnigoni et al., 2025).

Air sparging broadly refers to any technique involving the injection of air into fermenting must. For example, commercial configurations exist (e.g., Pulsair™) that introduce sequential jets of air through nozzles located on the sides or at the bottom of the fermentor. Depending upon bubble size and amount of air, this process mixes the fermenting must and may break the cap. For example, the so-called Pulsair™ technology has been shown to resuspend yeast that has settled to the bottom of fermentors, increasing O_2_ uptake and enhancing the rate of alcoholic fermentation (Saa et al., 2012). During alcoholic fermentation of Tempranillo, AirMixing™ was shown to enhance phenolic extraction, particularly color, by up to 21 % compared to traditional techniques such as pump overs and rack-and-returns (Parsec, n.d.; Guerrini et al., 2023). In contrast, Parnigoni et al. (2025) employed an air sparging system that delivered one-hour-long air sparging events at a rate that did not disrupt the cap. Their results indicated that air sparging at their reported rate lessened color intensity, lowered astringency, and decreased phenolic content in Petit Sirah and Pinot noir wines. While air sparging offers advantages over punch downs, such as decreased labor costs and consistent O_2_ delivery, the variability in techniques and outcomes across studies raises questions about its overall effectiveness.

Micro‑oxygenation (MiOx) is a winemaking technique that involves the controlled introduction of small amounts of O_2_ (e.g., 0.033 mg/L/month) into young red wines over time (Dykes, 2007). When applied to young red wines, MiOx reportedly decreases the perception of vegetal aromas and flavors (Vidal & Aagaard, 2008), stabilize color, and lower astringency, attributes commonly associated with traditional barrel aging. Indeed, MiOx was intentionally developed to replicate certain desirable characteristics of traditional barrel maturation, but in a shorter timeframe and at lower cost (Cejudo-Bastante et al., 2011). However, it achieves these effects without the infusion of oak-derived compounds such as oak lactones, volatile phenols, and furfuryl compounds (Gómez-Plaza & Cano-López, 2011; Pérez-Prieto et al., 2002).

Oxygenation is distinct from oxidation, which is associated with undesirable sensory outcomes such as color browning (Singleton & Cilliers, 1995), the degradation of fruity aromas (Lee et al., 2011), and negative chemical outcomes such as the depletion of wine antioxidants (Gómez-Plaza & Cano-López, 2011). The latter does not preclude that MiOx does not lead to oxidative effects. Ji et al. (2020) showed that MiOx promoted acetaldehyde-producing yeast populations in various red wines. However, these yeast populations did not persist in wines that contained adequate levels of SO_2_ (Ji et al., 2020). The chemical oxidation of ethanol to acetaldehyde is triggered by H_2_O_2_, either through direct reactions with ethanol (Wildenradt & Singleton, 1974) or more likely through the formation of HO• via the Fenton reaction (Elias & Waterhouse, 2010), which is a more powerful oxidizing agent than H_2_O_2_, capable of directly attacking ethanol (Kreitman et al., 2013). Both chemical pathways can be inhibited through the reaction between H_2_O_2_ and SO_2_. This indicates that applying MiOx to red wines with sufficient SO_2_ presents a lower risk of microbial and chemical oxidation due to the antimicrobial activity of SO_2_ (Ough & Cromwell, 1987), its capacity to bind acetaldehyde (Miao & Waterhouse, 2025), and its ability to scavenge H_2_O_2_ (Danilewicz, 2016). Although protective, the desired effects of MiOx may be precluded in the presence of SO_2_.

Conversely, macro‑oxygenation (MOX) applies larger doses of O_2_ (measured in mg O_2_/L must/day) during alcoholic fermentation. MOX, through techniques such as Pulsair™ and controlled gas injection, introduces O_2_ without disrupting the cap, potentially offering a balance between flavor development and phenolic preservation (Parks, 2006; Pettinelli et al., 2022). Compared to punch downs, MOX may yield wines with lower flavan-3-ol content; compared to air sparging, it may offer superior color and total phenolic retention (Parnigoni et al., 2025); and compared to passive or no cap management, it may decrease the risk of reductive aromas caused by yeast under hypoxic stress (Du Toit et al., 2006; Parnigoni et al., 2025).

Despite these possibilities, current research has not thoroughly evaluated the impact of continuous, controlled oxygen delivery during alcoholic fermentation on the resulting wine's chemistry and sensory properties. The control of the ORP also offers a possibility to fully automate sparging protocols during alcoholic fermentation of red wines. Therefore, the present study aims to evaluate the chemical and sensory effects of macro‑oxygenation, redox-controlled air sparging, and punch downs during alcoholic fermentation of Syrah wines, and their effect on the chemical and detailed phenolic composition of the resulting wines.

## 2 Materials and methods

### 2.1 Winemaking

Syrah grapes were harvested on November 6, 2024, from the Rancho Real Vineyard (Santa Maria AVA, CA, USA) and delivered to the Cal Poly Research Winery for processing the same day of harvest. Grapes were manually loaded into an Armbruster VST-F 3000 vibrating hopper and sorting table (Armbruster, Güglingen-Frauenzimmern, GER), and conveyed via belt to a RotoVib destemmer (Armbruster, Güglingen-Frauenzimmern, GER). Destemmed fruit was pumped with a positive displacement must pump (Waukesha Cherry-Burrell, Delavan, WI, USA) into twelve 110 L jacketed stainless-steel fermentors (Westec, Healdsburg, CA, USA). Each fermentor was filled with 83 kg of must and received 50 mg/L of SO_2_. Fermentors were randomly assigned to one of the four following treatments: punch downs (PD), air sparging (RedoxCon), macro‑oxygenation at a per-provider suggested dosage rate (MOX), and macro‑oxygenation at double the suggested dosage rate (2MOX). Each treatment was performed in triplicate. Three hours after SO_2_ addition, the fermentors received 60 g/hL of Go-Ferm Sterol Flash yeast nutrient, followed by 60 g/hL of Lalvin EC-1118 yeast (Lallemand, Montreal, Quebec, CAN), and subsequently mixed with a punch down tool (Vintner Vault, Paso Robles, CA, USA) for one minute. Double the suggested dosage rates were used for Go-Ferm Sterol Flash yeast nutrient and Lalvin EC-1118 yeast to avoid sluggish fermentation.

Each fermentor was connected to a glycol heating system (controlled to maintain 28 °C) and monitored using RC-5+ temperature probes (Elitech, San Jose, CA, USA) inserted horizontally through a hollow tube in the middle of each fermentor. EasyFerm Plus Ag/AgCl ORP sensors (Hamilton Company, Reno, NV, USA), housed in custom stainless-steel enclosures (Beringer Vineyards, Saint Helena, CA, USA), were inserted into each fermentor and connected via VP8 cables to redox control boxes built by MeshVines (Davis, CA, USA). Before use, the ORP sensors were calibrated using +271 mV ORP buffer and the ArcAir application (Hamilton Company) via a Wi 1G adapter.

PD consisted of manual 1 min punch downs twice daily using a punch down tool, with two punch downs replaced by full-volume rack-and-returns on day 3 of alcoholic fermentation to supply more O_2_ to yeast and homogenize temperature gradients at peak alcoholic fermentation. For RedoxCon, stainless-steel sparging spears with 2 μm pore size sinter elements (Must Machining and Fabrication, Napa Valley, CA, USA) were submerged and adjusted to lie 10 cm above the bottom of the fermentor, on the opposite side from the ORP probe. Air sparging (10 s bursts), was triggered automatically whenever the ORP dropped below −40 mV, controlled via redox control boxes and regulated to 7 L/min (a rate which only broke a small hole in the cap directly above the sinter element) using a Model 351 flow meter (Harris Products Group, Mason, OH, USA).

**Fig. 1:**
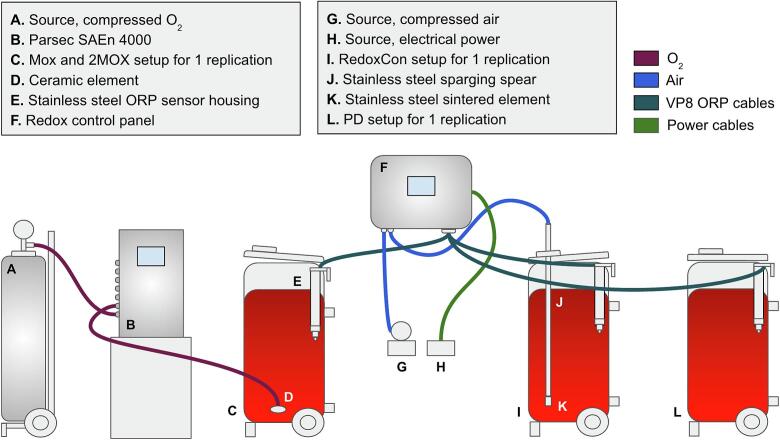
Experimental design during alcoholic fermentation of Syrah wines, from crush until pressing. Each stainless-steel fermentor (C, I, and L) represents *n* = 3 replicates per treatment. C presents both MOX and 2MOX treatments. O_2_ was supplied to MOX and 2MOX from a pressurized tank, and RedoxCon received air from a compressed air system.

The MOX and 2MOX treatments used 0.37 μm average pore size (Chiciuc et al., 2010) ceramic elements connected to a SAEn 4000 micro/macro‑oxygenation system (Parsec SRL, Florence, ITA), placed upward-facing at the fermentor base. Oxygen delivery was programmed via the SAEn 5000 online platform. MOX received 2 mg/L/day on day 1, 4 mg/L/day from days 2–4, and 1.3 mg/L/day from days 5–7. 2MOX received 4 mg/L/day on day 1, 8 mg/L/day from days 2–4, and 2.6 mg/L/day from days 5–7. The configuration of all treatments is shown in Fig. 1, and an estimation of bubble size geometry and physics of release is shown in Supplementary Fig. 1.

All treatments commenced on November 7, 2024 and concluded on November 13, 2024. All fermentors received 52 g/hL of Fermaid K and 15 g/hL diammonium phosphate (DAP) on day 2 (Vintner Vault, Paso Robles, CA, USA). After completion of maceration, free-run wine was drained by gravity from each fermentor's bottom valve (which will be referred to as pressing), then pumped using an air pump (Pumping Solutions Inc., Ontario, CA, USA) into two 20 L stainless-steel kegs (G4 Kegs, Tualatin, OR, USA). Lalvin VP41 malolactic bacteria (1 g/hL, Lallemand, Montreal, Quebec, CAN) were added to initiate malolactic fermentation (MLF), which occurred sequentially after completion of alcoholic fermentation to avoid the confounding effect of MLF on ORP (Parnigoni et al., 2025). The kegs were left at room temperature for approximately 30 days to allow for the completion of MLF. Once MLF was completed (malic acid <0.2 g/L), the kegs were moved to a cold room (15 °C), racked off gross lees, and subsequently dosed with SO_2_ to achieve a molecular SO_2_ concentration of 0.35 mg/L. Before bottling, the wines were racked off fine lees, pad filtered to 2 μm (Filtrox, St. Gallen, CHE) with an F10–20–30-40 inox filtration system with an inox pump (Enotecnica Pillan, Rampazzo, Vicentino, ITA) and re-adjusted to 0.35 mg/L molecular SO_2_ before bottling.

The wines were bottled using a custom bottle filler (XpressFill, San Luis Obispo, CA, USA) and sealed with a manual industrial cork stopper (Leo Victor, Italy) under technical corks (Diem 30, 49 mm height x 23.5 mm diameter, G3 Enterprises, Modesto, CA, USA). Each bottle was flushed with N_2_ (nitrogen gas) before being filled with wine, and Ar (argon gas) was used to displace any O_2_ in the headspace before corking. The finished bottles were then stored vertically and placed in a room set to 15 °C. After bottling, 50 mL samples of each replication were pipetted into pre-N_2_ sparged 50 mL glass ampoules (Wheaton Industries, Millville, NJ, USA), which were sealed using a blow torch (BernzOmatic, Worthington, OH, USA). The ampoules were then placed in an incubator (Thermo Fisher Scientific, Waltham, MA, USA) set to 38 °C and left undisturbed for 5 weeks, following an accelerated aging protocol (Casassa et al., 2023) that was previously validated for Pinot noir and Petite Sirah wines (Parnigoni et al., 2025).

### 2.2 Chemical analysis

#### 2.2.1 Fruit chemistry & physical composition

Fifteen clusters of Syrah grapes were randomly selected at harvest. From this, 200 randomly chosen berries were trimmed off the stems with scissors. Of these, 90 berries were selected, and their pedicels were removed by hand. The total weight of the selected berries was recorded using an Education ALF203 200 g digital scale (Thermo Fisher Scientific, Waltham, MA, USA). Each berry was sliced in half with an X-ACTO knife (Elmer's Products, Westerville, OH, USA), and the pulp was smeared away with a paper towel. The seeds were set aside and blotted dry. The total weight of the dried skins and seeds was recorded, and the number of seeds was counted. The liquid-to-solid ratio of the fruit was calculated by subtracting the sum of the total seed and skin weights from the total berry weight, then dividing by the sum of the total seed and skin weights. On average, the Syrah berries weighed 1.81 g and were composed of 8.8 % skins and seeds, and 91.2 % pulp. Each berry contained an average of 1.66 seeds (Supplementary Table 1).

#### 2.2.2 Brix and temperature monitoring during alcoholic fermentation

Brix (degrees) and temperature (°C) were measured daily during alcoholic fermentation with a DMA 35 (Anton Paar, Graz, AUT) from a 500 mL sample collected from the racking valve. All samples were centrifuged in an Eppendorf model 5415D microfuge (Eppendorf, Enfield, CT, USA) at room temperature and 15,000 g for 8 min. Samples undergoing active alcoholic fermentation were centrifuged following the same protocol, but at 2 °C. All analyses listed below (Sections 2.2.3 through 2.2.7) were performed using supernatant collected after centrifugation.

#### 2.2.3 Basic chemistry

The chemistry of the wines was measured with the same methods described for juice chemistry, with the additional analyses of acetic acid, lactic acid, free SO_2_, total SO_2_, and acetaldehyde, which were measured enzymatically or spectrophotometrically (Spica, Admeo, Angwin, CA, USA) using commercially available kits (Biosystems, Barcelona, ESP). Ethanol (% *v*/v) was measured by near-infrared spectroscopy using an Alcolyzer Wine M analysis system (Anton Paar, Graz, AUT).

#### 2.2.4 Spectrophotometric analysis

Color parameters, anthocyanins, tannins, polymeric pigments, and total phenolics were tracked throughout the winemaking process and during aging. Analyses were performed using a Cary 60 UV–Vis spectrophotometer with an 18-position auto-sampler (Agilent Technologies, Santa Clara, CA, USA). Anthocyanin concentrations were reported as mg/L malvidin-3-glucoside, while total phenolics were quantified as mg/L gallic acid equivalents. Polymeric pigments were measured using the Adams-Harbertson assay, following the method outlined by Harbertson et al. (2003). Tannin levels were assessed via protein precipitation and expressed in (+)-catechin equivalents (CE). Spectral scans and color evaluations (200 to 800 nm) were conducted in 1 mm quartz cuvettes using Cary WinUV Color software (Version 6.0, Startek Technology, Denver, CO, USA).

#### 2.2.5 Anthocyanins, anthocyanin-derived pigments, and flavonols

High-performance liquid chromatography with diode array detection (HPLC-DAD) was used to analyze wine samples throughout alcoholic fermentation, bottle aging, and following accelerated aging. Analyses were carried out using an Agilent 1100 series HPLC system equipped with a diode array detector (Agilent Technologies, Santa Clara, CA). Chromatographic separation was achieved using a gradient of two solvents: Solvent A (5 % formic acid in water) and Solvent B (methanol), delivered at a flow rate of 1 mL/min. The gradient program was as follows: 23 % B at 0 min, 26 % B at 5 min, 60 % B at 15 min, and 100 % B at 16 min. A 25 μL injection volume was used for each analysis. Separation was performed on an Agilent Zorbax Eclipse Plus C18 column (4.6 × 100 mm, 3.5 μm particle size; Agilent Technologies, Santa Clara, CA, USA) with a matching guard column, both maintained at 40 °C. Before injection, the column was equilibrated for 2 min at 23 % Solvent B. The DAD collected spectral data from 210 to 600 nm, with anthocyanins and flavonols quantified by measuring peak areas at 520 nm and 356 nm, respectively. All solvents were of HPLC grade and obtained from Thermo Fisher Scientific (Waltham, MA, USA).

Monomeric anthocyanins were quantified using a malvidin-3-glucoside chloride standard (Extrasynthèse, Lyon, FRA) and a calibration curve with R^2^ = 0.99. Pigment classes were grouped as follows: (1) malvidin-3-glucoside and its derivatives, (2) other glycosylated anthocyanins (including monoglucosides of delphinidin, cyanidin, petunidin, and peonidin), (3) acylated anthocyanins (such as acylated and coumaroylated monoglucosides), and (4) polymeric pigments and vitisins (including pyranoanthocyanins such as Vitisin A and B, and polymeric pigments). Flavonols were quantified using quercetin-3-glucoside (Sigma-Aldrich, St. Louis, MO, USA) as a reference standard, with calibration also showing R^2^ = 0.99. These compounds were classified into two categories: quercetin derivatives (e.g., quercetin-3-glucoside, quercetin-3-glucuronide, and quercetin aglycone) and other flavonols (e.g., myricetin, laricitrin, isorhamnetin, syringetin, kaempferol, and their derivatives).

#### 2.2.6 Monomeric flavan-3-ols

At designated intervals during aging, wine samples were analyzed using an Agilent 1260 Infinity II HPLC system coupled with an Agilent Ultivo mass spectrometer (Agilent Technologies, Santa Clara, CA, USA), operating with a scan range of *m*/*z* 100 to 1400. For each run, a 10 μL aliquot was injected onto an Agilent Zorbax Eclipse Plus C18 column (4.6 × 100 mm, 3.5 μm particle size; Agilent Technologies). The chromatographic separation utilized two mobile phases: (A) 0.1 % formic acid in water and (B) 0.1 % formic acid in acetonitrile, delivered at a flow rate of 0.8 mL/min. The column was maintained at a constant temperature of 25 °C. The solvent gradient was programmed as follows: 3 % B at 0 and 2 min, 6 % B at 10 min, 42 % B at 25 min, 100 % B at 30 min, and back to 3 % B at 32.5 min. Samples stored in the autosampler were held to 10 °C before analysis. Electrospray ionization (ESI) was performed in negative mode using Agilent's dual ESI source. The ion source parameters included a drying gas flow of 11.0 L/min at 325 °C, a nebulizer pressure of 35 psi, a capillary voltage of 3000 V, and a fragmentor voltage set to 130 V. Full-scan mass spectra were acquired across a range of *m*/*z* 100 to 2000. Prior to each analytical batch, the instrument was calibrated according to the manufacturer's standard protocol.

#### 2.2.7 Volatile compounds

Volatile compounds in the wine samples were extracted using stir bar sorptive extraction (SBSE) with polydimethylsiloxane (PDMS)-coated stir bars (Twister®, Gerstel, GER), following the methodology described by Marín-San Román et al. (2022). Extraction was conducted by immersing the Twister® stir bars directly into the wine samples and agitating at 1000 rpm for one hour at room temperature. After extraction, the volatiles were thermally desorbed using a thermal desorption unit (TDU2, Gerstel, GER) connected to a cooled injection system (CIS-4, Gerstel, GER), which was fitted with a liner containing 20 mg of Tenax TA®. Thermal desorption and gas chromatographic parameters followed the procedures outlined by Marín-San Román et al. (2022). The desorbed volatile compounds were separated using an Agilent 8890 gas chromatograph (GC) coupled to an Agilent 7000D triple quadrupole (QqQ) mass spectrometer (Agilent Technologies) operating in single quadrupole mode. Quantification was based on calibration curves generated from authentic standards prepared in synthetic wine (13.5 % ethanol, 5 g/L tartaric acid, pH 3.6, 20 mg/L SO_2_). Calibration standards (purity >95 %) included ethyl butyrate, ethyl isovalerate, isoamyl acetate, ethyl hexanoate, hexyl acetate, ethyl *n*-octanoate, ethyl decanoate, methyl salicylate, 2-phenylethyl acetate, ethyl cinnamate, geraniol, *cis*-rose oxide, linalool, β-citronellol, nerol, β-ionone, and *trans*-nerolidol. For compounds not covered by these standards, identification was based on mass spectral comparison with entries from the NIST library, and quantification was performed using γ-hexalactone as an internal standard equivalent.

### 2.3 Sensory analysis (modified pivot profile©)

A previously described modified Pivot © Profile rapid descriptive analysis (Stoffel et al., 2025) was performed using RedJade software (RedJade, Pleasant Hill, CA, USA). Panelists were given the option to rate wines as “more than the Pivot ©” or “less than the Pivot ©” (data recorded in binary as: “more than Pivot ©” = 1, “less than Pivot ©” = 0). Sensory attributes were determined by a group of 6 experienced tasters trained in sensory science prior to the formal sensory panel. Attributes and standards included orthonasal aromas and mouthfeel characteristics. Panelists were exposed to sensory standards (Supplementary Table 2) in a single 10 min training session. PD was selected as the Pivot© because of its significance as a cap management industry standard in commercial red winemaking. During this training session, aroma standards were passed from panelist to panelist in clear pear-shaped ISO glasses. Testing and evaluation procedures were also explained at this time. The panel consisted of 15 (*n* = 15) wine industry professionals (8 males and 7 females, 21 to 60 years of age).

Panelists completed formal sensory evaluation in isolated sensory booths under red light (Luna 3AO, 18:18 W, Zaniboni Lighting, Clearwater, FL, USA). Three-digit randomly coded wine samples (30 mL pour size) were delivered in clear pear-shaped ISO glasses through sliding doors. Replicates were evaluated individually, resulting in three sessions per treatment, for a total of nine evaluations. Panelists were instructed to expectorate all samples and rinse with water (Fiji, the Wonderful Company, Los Angeles, CA, USA) ad libitum. Plain, unsalted crackers (Nabisco, East Hanover, NJ, USA) were supplied for palate cleansing. Before training and formal sensory evaluation, the panel was approved by the California Polytechnic State University Institutional Review Board (IRB) under IRRB Protocol # 2020–058. Informed and signed consent to participate in the panel was obtained from each panelist prior to the training and evaluations.

### 2.4 Statistical analysis

Excel (Microsoft, Redmond, WA, USA) was used for data organization and supplementary tables. JMP (JMP Statistical Discovery LLC, Cary, NC, USA) was used for all analyses of variance (ANOVA), post hoc tests, and a variance decomposition table. ANOVA was used to test for statistical significance in differences among treatment means, while the Tukey-Kramer HSD (honestly significant difference) post hoc test was used to identify which treatment means differed significantly. Prism (GraphPad Software Inc., La Jolla, CA, USA) was used to create all figures except for Fig. 1, which was made using Google Slides (Google, Mountain View, CA, USA).

## 3 Results and discussion

### 3.1 Fermentation kinetics and evolution of the oxidation-reduction potential

**Fig. 2:**
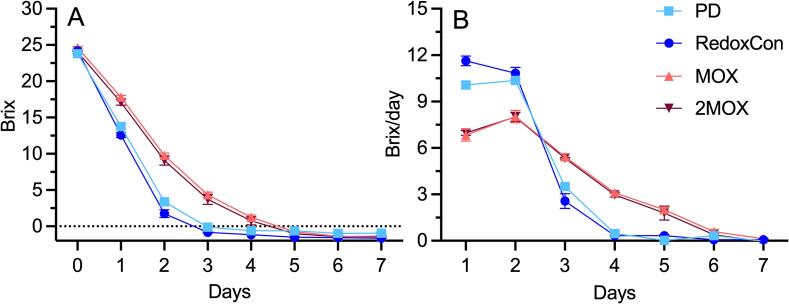
**A)** Evolution of sugar consumption (measured in Brix), and **B)** Brix consumed per day of alcoholic fermentation of Syrah wines from crush to completion of alcoholic fermentation. The horizontal dotted line in A) designates 0 Brix. Data points represent the mean of three replicates (*n* = 3), and error bars represent the standard error of the mean.

Syrah musts began alcoholic fermentation at 23.8 Brix. From this starting point, PD and RedoxCon reached <0 Brix on day 3, whereas MOX and 2MOX achieved <0 Brix on day 5 (Fig. 2). Moreover, PD and RedoxCon outperformed MOX and 2MOX in terms of Brix consumption rate, with 89 % of the Brix consumed by day 2 of alcoholic fermentation of PD and RedoxCon, compared to only 61 % for MOX and 2MOX. The shorter alcoholic fermentation times observed in PD and RedoxCon are likely due to the mixing and O_2_-dissolving effects of punch downs and sinter element-mediated air sparging, as previously reported (Parnigoni et al., 2025). MOX and 2MOX were not subject to the mixing or O_2_-dissolving effects of punch downs or air sparging seen in PD and RedoxCon. Supporting this explanation, prior research has shown that mixing during alcoholic fermentation resuspends solids, which in turn accelerates fermentation rates by *S. cerevisiae* in grape juice (Nienow et al., 2010).

**Fig. 3:**
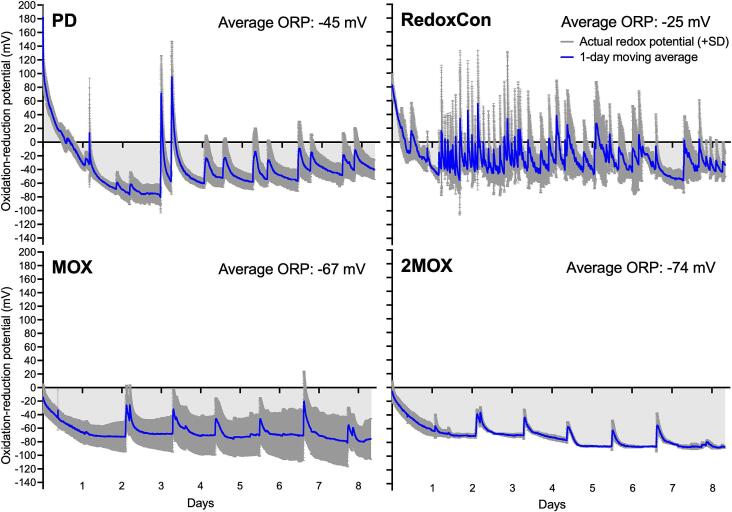
ORP of PD, RedoxCon, MOX, and 2MOX measured during alcoholic fermentation. Each graph represents an average of 3 replicates (n = 3). Blue lines represent the one-day moving average, while dark grey represents the standard deviation. Average ORP (mV) was calculated as the average of ORP values from the start to the end of alcoholic fermentation. (For interpretation of the references to color in this figure legend, the reader is referred to the web version of this article.)

The RedoxCon protocol was designed to maintain an ORP value above −40 mV, under the assumption that the S/H_2_S (elemental sulfur/hydrogen sulfide) redox couple will be 99.9 % in the oxidized state, that is, S (Nelson et al., 2022). ORP values above −40 mV were maintained in RedoxCon by 10 s air sparges triggered when redox values reached −40 mV (Fig. 3). The ORP of PD ranged from −80 mV at peak alcoholic fermentation to +100 mV, with peaks in the first two days being smaller (about +10 mV) compared to other treatments, coinciding with the onset and peak of alcoholic fermentation. The peaks became more pronounced, climbing +180 and + 160 mV due to rack-and-returns on day 3. After rack-and-returns on day 3, the ORP peaks became more consistent, oscillating between +20 and + 30 mV. The average ORP for MOX and 2MOX wines, calculated from the beginning to the end of treatment, was −67 mV and − 74 mV, respectively. The once daily peaks observed in MOX and 2MOX (increases ranging from +20 mV to +50 mV) are a result of sampling events, not sparging events. These peaks, however, were not observed in PD and RedoxCon, possibly due to a higher fraction of oxidized metals and reduced availability of nucleophiles.

Overall, RedoxCon received 147.6 mL O_2_/L must (or a total of 12.3 L O_2_ per tank) from the start to the end of treatment. MOX, on the other hand, received only 12.05 mL O_2_/L must (or a total of 1 L O_2_ per tank), and 2MOX received 24.1 mL O_2_/L must (or a total of 2 L O_2_ per tank). Despite receiving more oxygen than MOX, 2MOX maintained lower redox potentials, suggesting that increased O_2_ (i.e., one extra L of O_2_ per tank), stimulated yeast metabolism, possibly promoting the release of nucleophilic compounds like GSH, then lowering ORP (Danilewicz, 2012; Killeen et al., 2018; Nelson et al., 2025).

Although RedoxCon received 1125 % and 513 % more O_2_ than MOX and 2MOX (based on O_2_/L must), respectively, the bubbles delivered in MOX and 2MOX were estimated to be smaller and fewer in quantity (Supplementary Fig. 1). We estimated that the average bubble volume from a 2 μm pore size sinter element set to deliver air at 7 L/min was 140 % larger than the average bubble from a 0.37 μm pore size ceramic element set to 8 mg/L/day (OpenAI, San Francisco, CA, USA, Supplementary Table 3). Despite quantity, these finer bubbles in MOX and 2MOX might have dissolved as efficiently as the larger, more rapidly injected bubbles in the RedoxCon treatment. Differences in the solubility of air and O_2_ in fermenting must and limitations in visually detecting bubbles in red wine (hence, the use of white wine in Supplementary Fig. 1) due to phenolics and other red wine components, which may also affect air and O_2_ solubility, are factors that warrant further investigation.

### 3.2 SO_2_ and glutathione

In the present study, 50 mg/L SO_2_ was added at crush to inhibit PPO activity and control for the impacts of enzyme-mediated (biological) oxidation on ORP. No significant differences in free SO_2_ were observed at pressing (Supplementary Table 4). However, total SO_2_ levels were 116 % and 33 % higher in MOX compared to RedoxCon and PD, 147 % and 52 % higher in 2MOX compared to RedoxCon and PD, and 62 % higher in PD compared to RedoxCon at pressing (*p* < 0.0001). The larger total SO_2_ seen in MOX, 2MOX, and PD is likely attributed to the release of SO_2_ from yeast in unfavorable conditions (Berry & Watson, 1987) and the non-volatile nature of the SO_2_-acetaldehyde adduct (Liu & Pilone, 2000), while the lower concentration in RedoxCon is attributed to SO_2_ stripping (in the absence of acetaldehyde) by CO_2_ and vigorous air sparging (Henderson, 2009).

**Fig. 4:**
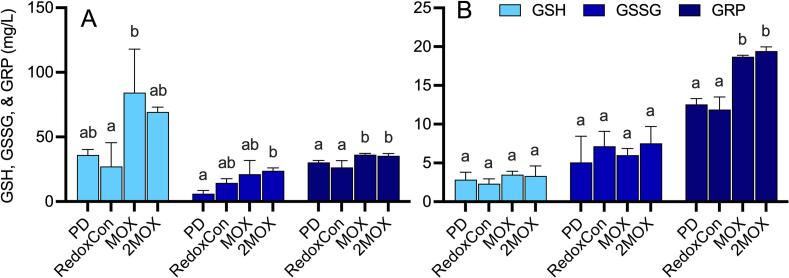
Reduced glutathione (GSH), glutathione disulfide (GSSG), and grape reaction product (GRP) at **A)** pressing and **B)** 3 months bottle aging, in mg/L. Significant letters are provided, indicating statistical differences in GSH, GSSG, and GRP between treatments at each time point using the Tukey-Kramer HSD test and *p* < 0.05.

GSH was 210 % higher in MOX than in RedoxCon (*p* = 0.0203), and glutathione disulfide (GSSG) was 293 % higher (*p* = 0.0238) in 2MOX than in PD at pressing. No differences were observed in GSH or GSSG at 3 months of bottle aging. GRP, which is formed through the reaction of the quinone of caftaric acid with glutathione (Singleton et al., 1985) was found to be 54 % and 46 % higher in MOX than in PD and RedoxCon, and 59 % and 51 % higher in 2MOX than in PD and RedoxCon at pressing (*p* = 0.0008). After 3 months of bottle aging (*p* < 0.0001), GRP was 49 % and 57 % higher in MOX than in PD and RedoxCon, and 55 % and 63 % higher in 2MOX than in PD and RedoxCon. The results presented in Fig. 4 suggest that GSH initially was better preserved in MOX and 2MOX. These were the ferments that happened to receive the least oxygen. Fig. 4 also suggests that GRP formation was highly dependent on the initial preservation of GSH during alcoholic fermentation. Conversely, in PD and RedoxCon, GSH may have initially reacted with caftaric acid quinones to form GRP, which could then have undergone further oxidation to generate new quinones, although higher GRP levels were not recorded in these wines. This discrepancy may be explained by the production of a secondary quinone and its reaction to an additional glutathione molecule, resulting in the formation of grape reaction product 2, known as GRP2 (Cejudo-Bastante et al., 2010; Cheynier & Van Hulst, 1988). GRP2 was not quantified in the present study; it remains a subject of interest for future research.

### 3.3 Basic chemistry of the wines

No significant differences were observed in ethanol level and pH at pressing between treatments (Table 1). However, titratable acidity (TA) was lower in PD than in RedoxCon, MOX, and 2MOX (*p* = 0.0052). Acetic acid was significantly higher in PD and RedoxCon than in MOX and 2MOX at pressing (*p* < 0.0001) and bottling (*p* < 0.0001). *S. cerevisiae*, other yeasts, and lactic acid bacteria likely played a role in the production of acetic acid in PD and RedoxCon wines (Guindal et al., 2023; Rachwał & Gustaw, 2024). However, acetic acid levels were generally low and well below their identification threshold (Macías et al., 2012) and thus unlikely to bear sensory relevance. Acetaldehyde (Supplementary Table 4) was higher in MOX and 2MOX than in PD and RedoxCon at pressing (*p* < 0.0001) and bottling (*p* < 0.0001). Although acetic acid concentrations increased slightly from pressing to bottling due to MLF, acetaldehyde levels decreased by an average of 82 % across all treatments. This was likely due to binding with SO_2_ (dosed at bottling) or consumption by malolactic bacteria prior to bottling (Osborne et al., 2006). Bound acetaldehyde, which the enzymatic method employed in the present study may not include, must be quantified in future studies to confirm the latter, as acetaldehyde bound to SO_2_ (hydroxysulfonate) is non-volatile (Liu & Pilone, 2000) and therefore would not contribute sensorially.

**Table 1:** Basic chemistry of Syrah wines at pressing and bottling.

Time Point	Treatment	Ethanol (% v/v)	pH	Titratable acidity (g/L)	Lactic acid (g/L)	Malic acid (g/L)	Acetic acid (g/L)
Pressing	PD	14.4 a	3.48 a	7.60 a	0.14 a	2.30 ab	0.13 a
	RedoxCon	14.2 a	3.46 a	8.16 b	0.15 a	2.23 a	0.15 a
	MOX	14.5 a	3.45 a	8.15 b	0.12 a	2.41 ab	0.06 b
	2MOX	14.2 a	3.45 a	8.22 b	0.13 a	2.53 b	0.04 b
	*p*-value	0.1189	0.2595	**0.0052**⁎	0.3423	**0.0146**	**< 0.0001**

Bottling	PD	14.6 a	3.64 a	7.51 a	1.34 ab	0.03 ab	0.31 a
	RedoxCon	14.3 ab	3.57 b	7.39 a	1.30 a	0.02 a	0.34 a
	MOX	14.5 ab	3.52 bc	7.55 a	1.39 ab	0.04 b	0.13 b
	2MOX	14.2 b	3.51 c	7.64 a	1.45 b	0.04 b	0.09 b
	*p*-value	**0.0380**	**0.0003**	0.8726	**0.0203**	**0.0191**	**< 0.0001**

⁎ Bold values indicate significant differences between treatments for the Tukey-Kramer HSD test and *p* < 0.05.

### 3.4 Color

**Fig. 5:**
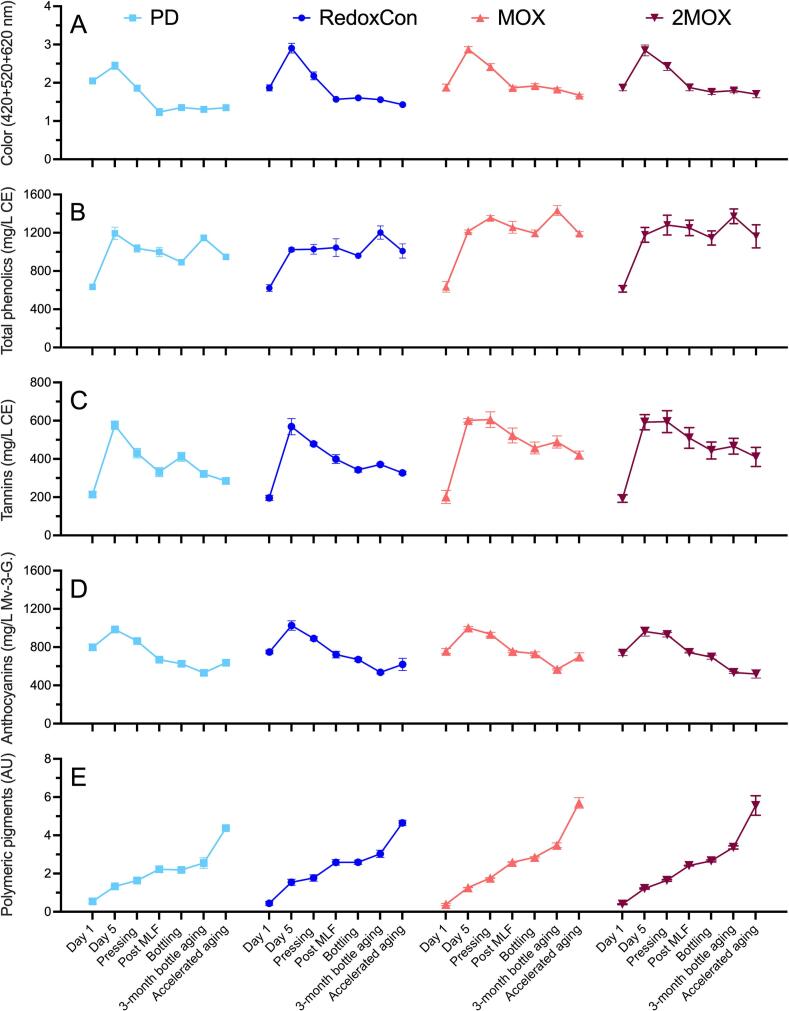
Phenolic composition of Syrah wines throughout winemaking and accelerated aging. **A)** wine color as the sum of absorbance values at 420 nm, 520 nm, and 620 nm, **B)** total phenolics, **C)** tannins, **D)** anthocyanins, and **E)** polymeric pigments.

No statistical differences in color (420 + 520 + 620 nm absorbances) among treatments were found during alcoholic fermentation (Fig. 5A). However, at pressing, 2MOX and MOX had 31 % and 30 % more color than PD (*p* = 0.0102). Similar trends were observed subsequently at post MLF (*p* = 0.0004) and bottling (*p* = 0.0001). After accelerated aging (*p* = 0.0027) and 3 months bottle aging (*p* < 0.0001), MOX and 2MOX exhibited higher color intensity than both PD and RedoxCon. MOX and 2MOX consistently contained more color than PD and RedoxCon, and RedoxCon had more color than PD after alcoholic fermentation through accelerated aging. These findings are supported by full visible spectra-scan data taken from 200 to 800 nm (Supplementary Fig. 2) and the actual aspects of the wines after bottling and accelerated aging (Supplementary Fig. 3).

Fig. 5A suggest that color peaked in all treatments on day 5 of alcoholic fermentation and declined steadily until SO_2_ was added at the post MLF time point. A slight rebound occurred at bottling (except in 2MOX), followed by another decline in color after accelerated aging (except in PD). The loss of color after day 3 is likely due to the reabsorption of anthocyanins and color compounds by lees and other fermentation solids (Mazauric & Salmon, 2006). The dramatic color loss in all treatments between pressing and post MLF is likely due to anthocyanin bleaching by SO_2_ (Somers & Evans, 1977) and the formation of tawny-colored acetaldehyde-mediated anthocyanin-tannin condensation products called polymeric pigments (Somers, 1971). Previous studies have shown that wines made with no-cap management protocols have similar color profiles as those made with punch downs, suggesting that higher liquid-solid contact during cap management does not always correlate with higher color extraction (Casassa et al., 2024). However, in this study, MOX and 2MOX wines retained significantly more color than PD and RedoxCon, implying that physical cap intervention lessened color retention in Syrah wines, likely a result of higher pH values and the rapid onset of ethanol accumulation due to faster alcoholic fermentation times (Fig. 2) and subsequent precipitation of anthocyanins in less aqueous environments (Gull et al., 2022).

### 3.5 Phenolic compounds

Distinct differences in total phenolic composition emerged between MOX/2MOX and PD/RedoxCon by the end of alcoholic fermentation. On day 5, RedoxCon wines contained 14 %, 16 %, and 13 % fewer total phenolics than PD, MOX, and 2MOX (*p* = 0.0066). By pressing (*p* = 0.0003), total phenolics were 31 % higher in MOX than in PD, and 32 % higher than in RedoxCon. Total phenolics in 2MOX were also significantly higher than in PD and RedoxCon (by 24 % and 25 %, respectively) at pressing. Similar trends in total phenolics were observed at post MLF and bottling between MOX/2MOX and PD/RedoxCon wines. However, after accelerated aging, the total phenolic concentration in RedoxCon no longer differed from MOX and 2MOX. After accelerated aging (*p* = 0.0087), PD wines had 21 % and 19 % fewer phenolics than MOX and 2MOX wines, respectively. The application of MOX and, to a lesser extent, air sparging during alcoholic fermentation resulted in better total phenolic retention than PD after aging (Fig. 5B).

Tannins (Fig. 5C) were only slightly higher in MOX and 2MOX than in PD and RedoxCon from pressing through accelerated aging, except at bottling. Additionally, flavan-3-ols (Supplementary Fig. 4), were statistically indistinguishable among treatments at pressing and accelerated aging. Casassa et al. (2024) found that tannin extraction was initially similar in Cabernet Sauvignon wines made with no cap management. In the latter stages of alcoholic fermentation, however, extraction continued in punch down wines, while it plateaued in no cap management wines. Shorter maceration times in the present study may account for the similar tannin levels in MOX, 2MOX, PD, and RedoxCon treatments; however, the slightly higher tannin concentrations in MOX and 2MOX reflect higher temperatures in the cap, which were left undisturbed by gentle O_2_ delivery. A study with Barbera suggests that the retention of tannins may be more important than the quantity of tannins extracted (Bosso et al., 2011). In the present study, 100 % of the maximum concentration of tannins in PD and RedoxCon, and 99.4 % and 99.5 % of the maximum concentration of tannins in MOX and 2MOX were extracted by day 5. Tannins were found in the lowest concentrations after accelerated aging in all treatments. PD wines lost 51 % of their tannins between day 5 and accelerated aging, while RedoxCon lost 43 %, MOX lost 30 %, and 2MOX lost 31 %. These results support the hypothesis by Bosso et al. (2011) that gentler cap management protocols specifically improve long-term tannin retention.

Anthocyanins (Fig. 5D) differed only at the post MLF timepoint (*p* = 0.0472) and at bottling (*p* = 0.0397), with MOX showing higher concentrations than PD at both stages. Previous research has indicated that different cap management techniques (e.g., punch downs vs. no punch downs) had no significant effect on the concentration of anthocyanins in Grenache wines (Stoffel et al., 2023). The same study found that temperature significantly influenced anthocyanin content. However, temperature was a controlled variable in the present experiment (Supplementary Fig. 5). No significant differences in polymeric pigments (Fig. 5E) were observed at pressing. By bottling (*p* < 0.0001), MOX, 2MOX, and RedoxCon wines had 30 %, 21 %, and 18 % more polymeric pigments than PD, respectively. After accelerated aging (*p* = 0.0014), MOX and 2MOX remained significantly higher in polymeric pigment content than both PD and RedoxCon. We hypothesize that the elevated levels of anthocyanins and tannins in MOX and 2MOX contributed to the observed increases in polymeric pigments (Singleton & Trousdale, 1992). Supplementary Figs. 6 and 7 provide HPLC support for higher concentrations of anthocyanins and flavonols (which are, according to Boulton (2001), phenolic compounds responsible for augmenting color through copigmentation) in MOX and 2MOX compared to PD and RedoxCon wines.

### 3.6 Volatile composition of the wines

Esters, selected terpenes, and norisoprenoids were quantified at pressing and during accelerated aging using GC–MS. Odor activity values (OAV) were calculated by dividing the concentration of a compound (μg/L) by the published odor recognition threshold (μg/L) for each compound (Ferreira et al., 2000). Aromas with an OAV ≥ 1 are considered to contribute to the sensory profile of a given wine.

**Fig. 6:**
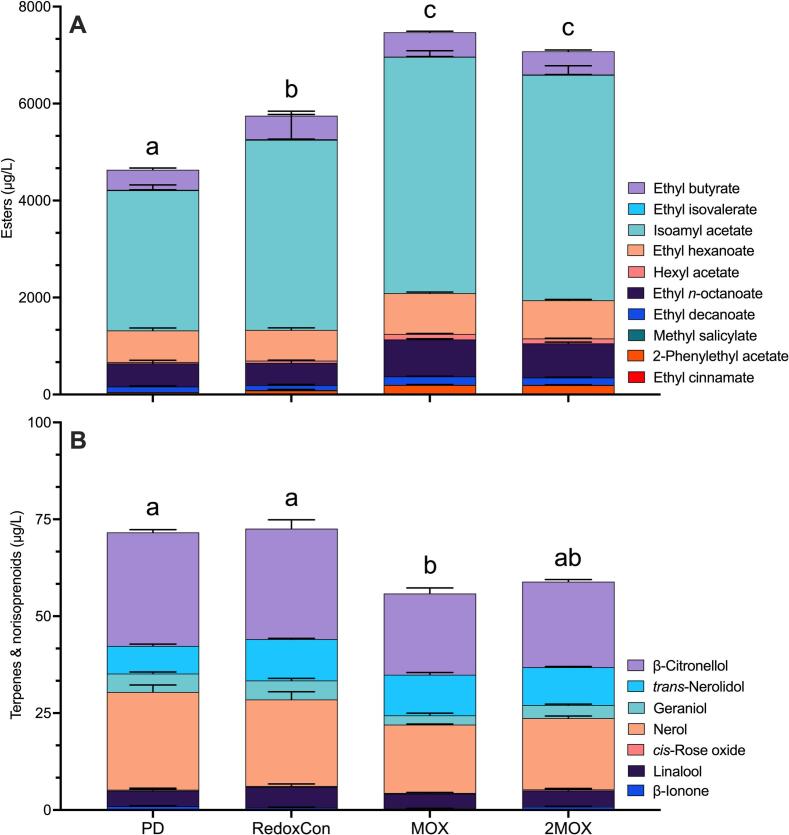
**A)** Esters, and **B)** terpenes and norisoprenoids at pressing. Significant letters are provided, indicating statistical differences in total esters and the sum of terpenes and norisoprenoids between treatments using the Tukey-Kramer HSD test and *p* < 0.05.

At pressing, the sum of all esters was higher in the MOX and 2MOX treatments compared to RedoxCon and PD wines, with PD wines containing the least (Fig. 6). Continuous and progressive oxygen delivery in MOX and 2MOX did not break the cap, as visually confirmed. However, cap disturbance expectedly occurred in the PD treatment. Still, this mechanical action unlikely stripped esters to the extent of vigorous bubbling observed in RedoxCon wines. Indeed, this observation aligns with recent work by Parnigoni et al. (2025), which found significantly higher levels of esters in wines made without cap management compared to those made with cap management (e.g., punch downs, pump overs, air sparging, and N_2_ sparging). Contrastingly, the production of yeast-derived acetate esters has been reported to increase under oxidative conditions (Valero et al., 2002). We suggest that the increase in acetate esters due to O_2_, however, is likely overshadowed by the stripping effects of CO_2_.

The following esters had OAVs ≥1 at the time of pressing: ethyl butyrate, ethyl hexanoate, ethyl isovalerate, ethyl *n*-octanoate, hexyl acetate (only in MOX and 2MOX), and isoamyl acetate. Among these, ethyl *n*-octanoate and isoamyl acetate had the highest OAVs, making them the most dominant sensorially. Specifically, ethyl *n*-octanoate (*p* < 0.0001) was 60 % and 66 % higher in MOX (OAV = 152.38) compared to PD (OAV = 95.39) and RedoxCon (OAV = 91.63), and 48 % and 54 % higher in 2MOX (OAV = 141.30) than in PD and RedoxCon. Isoamyl acetate (*p* = 0.0003) was 69 %, 61 %, and 18 % higher in MOX (OAV = 162.47), 2MOX (OAV = 154.96), and RedoxCon (OAV = 130.78) than in PD (OAV = 96.40). The OAVs of hexyl acetate (*p* < 0.0001) exceeded 1 in MOX (OAV = 1.12) and 2MOX (OAV = 1.00), but not in PD (OAV = 0.29) or RedoxCon (OAV = 0.49). Although an arbitrary value, the OAV of hexyl acetate might have impacted the sensory profiles of MOX and 2MOX but not PD and RedoxCon wines.

In contrast, terpenes and norisoprenoids were most abundant in PD and RedoxCon wines, and least abundant in MOX and 2MOX wines. This trend has been observed previously by Parnigoni et al. (2025), who found that volatiles, such as vitispirane and *trans*-1,8-terpin (Tarko et al., 2020), increase as wine undergoes oxidation. Additionally, the physical mixing of punch downs and air sparging events in PD and RedoxCon may have increased grape solid:liquid contact, which has been found to encourage the extraction of terpenes into must (Palomo et al., 2006). The only volatile with an OAV > 1 was β-ionone (*p* = 0.0110), which was 149 % and 115 % higher in PD (OAV = 10.97) and 2MOX (OAV = 9.48) than MOX (OAV = 4.41). β-Ionone likely contributed violet and dark berry aromas (Rutan et al., 2014) to all wines in the present study.

### 3.7 Sensory analysis by pivot© profile

**Fig. 7:**
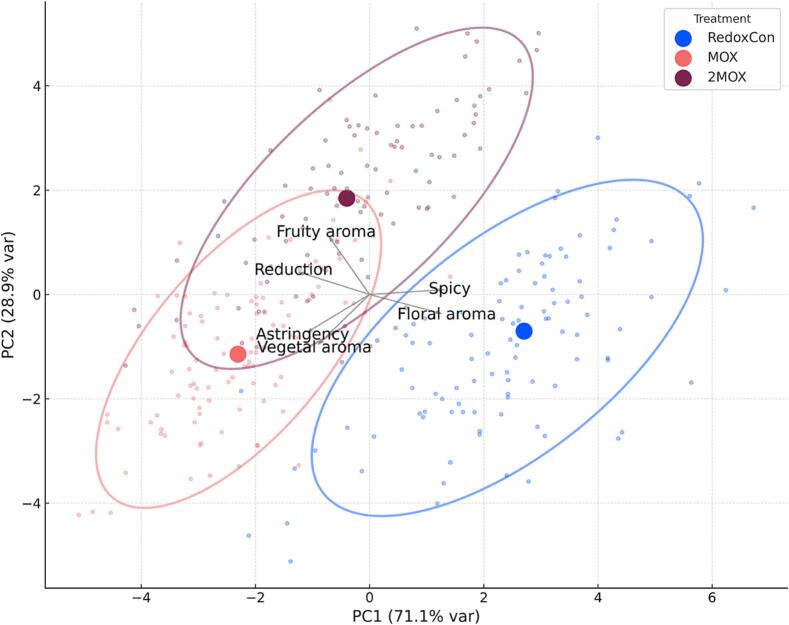
Principal component analysis and confidence ellipses of Pivot© Profile data for Syrah wines evaluated by sensory panel (n = 15).

A convened sensory panel (*n* = 15) was trained to identify six relevant sensory attributes in the wines: fruit aroma, spice aroma, floral aroma, vegetal aroma, reduction aroma, and the tactile sensation of astringency. Data was collected using a modified Pivot© Profile sensory method (Stoffel et al., 2025), under red light to eliminate color bias. PD, the most commercially standard cap management technique, was chosen to be the Pivot©. Fig. 7 displays the principal component analysis (PCA) plot of the Pivot© Profile results. A variance decomposition table (data not shown) found that 58.7 % of the sensory variance was due to treatment, whereas 41.3 % was due to panelist disagreement. Fisher's exact test (*p* < 0.05) is available in Supplementary Table 5, and a frequency of citation plot is shown in Supplementary Fig. 8.

Fisher's exact test suggested that RedoxCon wines were perceived to have less reduction aromas (*p* < 0.05) than MOX and 2MOX. Otherwise, no statistically significant differences in the wines were captured by the sensory panel. RedoxCon wines were characterized by spice and floral aromas. These sensory notes align with the fact that RedoxCon wines had more total terpenes and norisoprenoids relative to MOX wines. MOX wines were sensorially more astringent than RedoxCon wines, whereas 2MOX wines were characterized by fruit aroma, though they were also comparatively more astringent than RedoxCon wines. Importantly, there was an overlap between the ellipses of MOX and 2MOX wines, indicating that these two wines were similar from a sensory standpoint, sharing vegetal, fruity, and reductive aroma characteristics, as well as astringent mouthfeel. Conversely, the ellipse of RedoxCon wines did not superimpose, suggesting MOX and 2MOX were sensorially different, with RedoxCon wines characterized more by fruity and floral aromas.

## 4 Conclusions

This study explored the chemical effects of macro‑oxygenation and redox-controlled air sparging during alcoholic fermentation of cool climate Syrah wines from the Central Coast of California. The results show that macro‑oxygenation, redox-controlled air sparging, and punch down cap management significantly altered the chemical composition of Syrah wines. MOX was found to be an effective method for preserving the natively high phenolic composition of cool-climate Syrah wines, as well as color, and esters, compared to wines made with redox-controlled air sparging and punch downs. Furthermore, MOX and redox-controlled air sparging systems are automatable, offering potential cost savings, and in the case of air sparging configurations, a possible method to soften astringency in the finished wines. Perhaps more remarkably, we report that these cool-climate Syrah wines were able to accommodate oxygen addition rates during alcoholic fermentation that varied almost 11-fold (1125 %) in some cases. Given the magnitude of this range, it is noteworthy that its extremes, such as RedoxCon and MOX treatments, produced wines with much smaller variations in phenolics, albeit with different chemical and sensory characteristics.

## Abbreviations

**Table:** 

Ag/AgCl	silver/silver chloride
ANOVA	analysis of variance
Ar	argon gas
CO_2_	carbon dioxide
Cu^+^	cuprous ion
Cu^+2^	cupric ion
DAD	diode-array detector
DAP	diammonium phosphate
ESI	electrospray ionization
Fe^+2^	ferrous ion
Fe^+3^	ferric ion
GC	gas chromatography
GRP	grape reaction product
GSH	reduced glutathione
GSSG	glutathione disulfide
HO•	hydroxyl radical
HOO•	hydroperoxyl radical
HPLC	high-performance liquid chromatography
HSD	honestly significant difference
H_2_O	water
H_2_O_2_	hydrogen peroxide
H_2_S	hydrogen sulfide
IRB	internal review board
M.I.	modulated injection
MiOx	micro‑oxygenation
MLF	malolactic fermentation
MOx	macro‑oxygenation
MS	mass spectrometry
mV	millivolts
NIST	National Institute of Standards and Technology
N_2_	nitrogen gas
OAV	odor activity value
ORP	oxidation-reduction potential
O_2_	oxygen
PCA	principal component analysis
PD	punch down
PDMS	polydimethylsiloxane
PPO	polyphenol oxidase
QqQ	triple quadrupole
RedoxCon	redox-controlled air sparging
ROS	reactive oxygen species
S	elemental sulfur
SBSE	stir bar sorptive extraction
SO_2_	sulfur dioxide
TA	titratable acidity

## CRediT authorship contribution statement

**William Jordan Wright:** Writing – original draft, Project administration, Formal analysis, Data curation. **Sean Kuster:** Resources, Methodology, Investigation, Data curation. **Biljana Petrova:** Methodology, Formal analysis, Data curation. **Jesus Villalobos:** Resources, Investigation. **James Nelson:** Supervision, Resources. **Robert Coleman:** Writing – review & editing, Resources, Investigation. **Luis Federico Casassa:** Writing – review & editing, Validation, Supervision, Investigation, Funding acquisition, Data curation.

## Funding

No external funding was received for this study. E. & J. Gallo Winery (Healdsburg, California, USA) and Treasury Wine Estates (St. Helena, California, USA) are thanked for their donation of fruit and logistical support, respectively.

## Declaration of competing interest

The authors declare that they have no known competing financial interests or personal relationships that could have appeared to influence the work reported in this paper.

## Data Availability

Data will be made available on request.

## Appendix A Supplementary data

Supplementary material Image 1
