# Supplementary material for: Chemical and sensory effects of macro-oxygenation and air sparging coupled with oxidation-reduction potential (ORP) monitoring in Syrah wines
Source: Food Chem X. 2025 Oct 4;31:103135. doi: 10.1016/j.fochx.2025.103135 (PMC12538412; doi:10.1016/j.fochx.2025.103135)
Supplement: Supplementary file 1 — Supplementary material [file mmc1.docx]

**Supplementary Material**

| **Supplementary Table 1.** Berry composition of Syrah grapes at crush. Values represent the mean of three sample replicates; each composed of 30 berries (n = 90). | | | | | | |
| --- | --- | --- | --- | --- | --- | --- |
| Time point | Total weight per berry (g) | Skin weight per berry (g) | Seed weight per berry (g) | Pulp weight per berry (g) | Seeds per berry | Liquid: solid ratio |
| Crush | 1.81 | 0.11 | 0.05 | 1.65 | 1.66 | 10.12 |

| **Supplementary Table 2.** Detailed composition of the sensory standards used during the modified Pivot© Profile training and formal evaluation sessions of Syrah wines. | |
| --- | --- |
| Sensory attribute | Standard composition |
| Fruit | 10 mL blackberry syrup & 2 mL cherry syrup, Torani, USA |
| Floral | 5 mL violet syrup, Le Sirop de Monin, FRA |
| Spice | ½ teaspoon ground black pepper, First Street, Smart & Final, USA |
| Reduction | 200 μL hydrogen sulfide water, LabChem, (Cat#: LC154701), USA |
| Vegetal | 5 g chopped stems from seedless green grapes, Smart & Final, USA |
|  | 5 g chopped fresh poppy stems and leaves |
| Astringency | Definition provided to panelists |
| All standards were prepared in 400 mL of base wine (2023 Kirkland Cabernet Sauvignon). | |

| **Supplementary Table 3.** Estimated bubble volume and standard deviations (SD) measured by AI from images (n = 1,000) of air diffused via 2 μm sinter element set to 7 L/min and oxygen diffused via 0.37 μm pore size MOX element set to 8 mg/L/day in 15% v/v ethanol Viognier white wine. | | |
| --- | --- | --- |
| Treatment | Bubble volume (mm^3^) | SD bubble volume |
| RedoxCon | 0.60 | 0.07 |
| MaOX | 0.25 | 0.01 |

| **Supplementary Table 4.** Free and total sulfur dioxide of Syrah wines at pressing and bottling, in mg/L. | | | | |
| --- | --- | --- | --- | --- |
| Time point | Treatment | Free sulfur dioxide (mg/L) | Total sulfur dioxide (mg/L) | Acetaldehyde (mg/L) |
| Pressing | PD | 6.67 a | 17.33 a | 25.33 a |
|  | RedoxCon | 2.67 a | 10.67 b | 25.00 a |
|  | MOX | 5.67 a | 23.00 c | 31.33 b |
|  | 2MOX | 5.67 a | 26.33 c | 32.33 b |
|  | *p*-value | 0.2304 | **< 0.0001*** | **< 0.0001** |
| Bottling | PD | 11.67 a | 16.33 a | 3.00 a |
|  | RedoxCon | 8.00 a | 8.67 a | 2.00 a |
|  | MOX | 10.33 a | 13.00 a | 7.00 b |
|  | 2MOX | 8.67 a | 14.67 a | 8.33 b |
|  | *p*-value | 0.4424 | 0.0934 | **< 0.0001** |
| *Bold values indicate significant differences between treatments for the Tukey-Kramer HSD test and *p* < 0.05. | | | | |

| **Supplementary Table 5.** Significance by cell (Fisher's exact test). | | | | | | |
| --- | --- | --- | --- | --- | --- | --- |
| Treatment | Fruit aroma | Floral aroma | Spice aroma | Vegetal aroma | Reduction aroma | Astringency |
| RedoxCon | < | > | > | > | **<*** | < |
| MOX | < | < | < | > | > | > |
| 2MOX | > | < | < | < | > | < |
| *Bold values are significant at the level alpha = 0.05 | | | | | | |


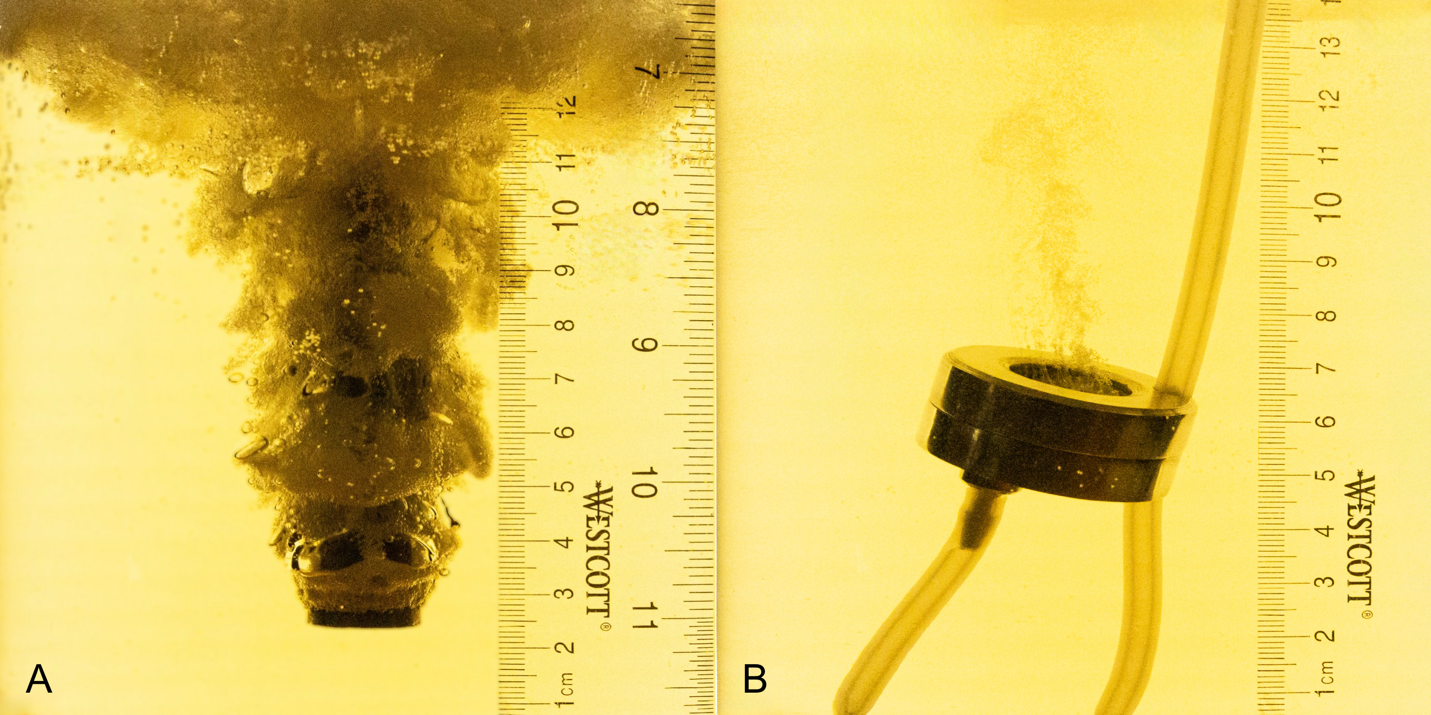


**Supplementary Figure 1.** Bubble size geometry and physics of release, demonstrated in a 15% v/v ethanol Viognier white wine for clarity. A) Presents a 2 μm pore size sinter element diffusing air at a rate of 7 L/min, and B) shows a Parsec 0.37 μm average pore size ceramic element diffusing oxygen at a rate of 8 mg/L/day.

**Supplementary Figure 2.** Spectrophotometric scans from 200 nm to 800 nm, measured as absorbance units (AU).


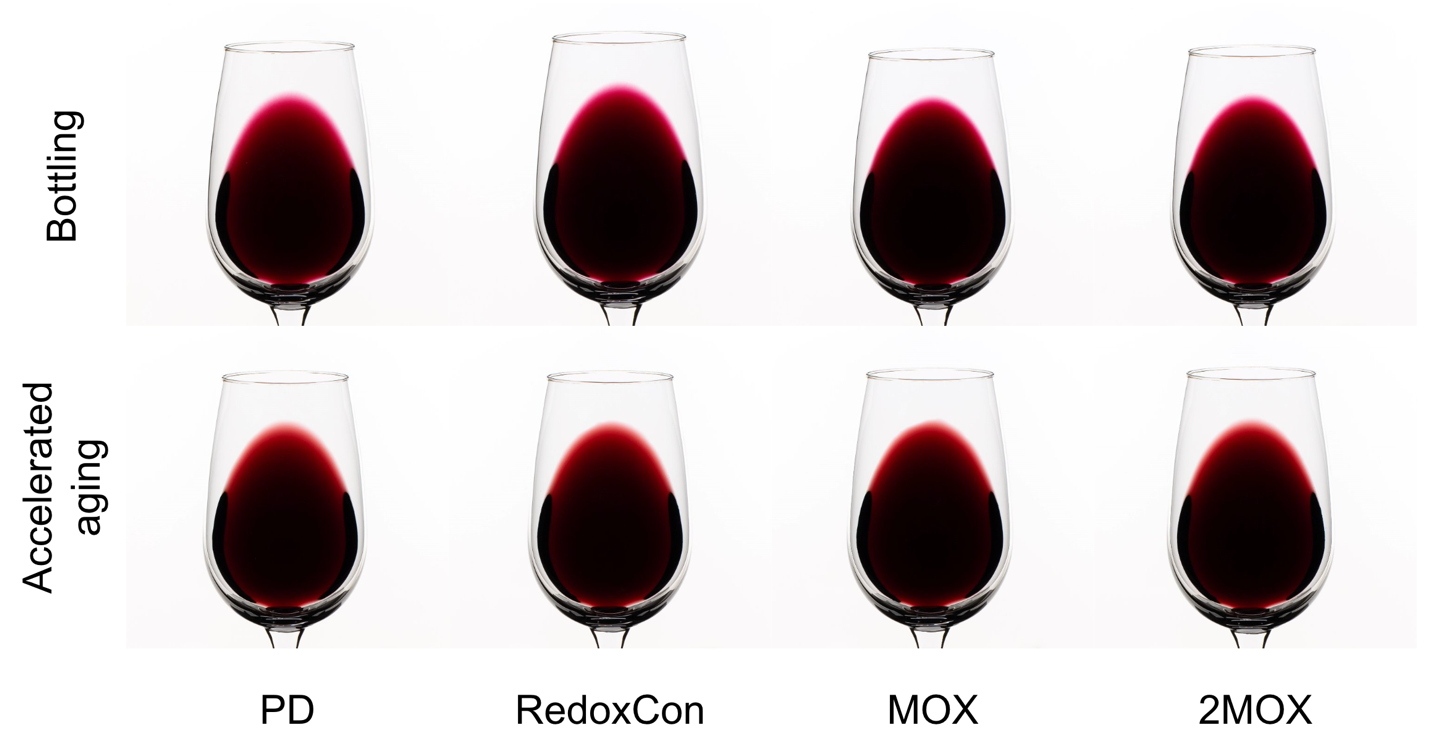


**Supplementary Figure 3.** Aspects of Syrah wines at bottling and accelerated aging.





**Supplementary Figure 4.** Monomeric flavan-3-ols at pressing and monomeric, dimeric, trimeric, and sulfonated flavan-3-ols at accelerated aging in Syrah wines, expressed as catechin equivalents. Significant letters are provided, indicating statistical differences in monomeric and total flavan-3-ols between treatments at pressing and accelerated aging, respectively, using the Tukey-Kramer HSD test and p < 0.05.

**Supplementary Figure 5.** Temperature of Syrah wines during alcoholic fermentation (ºC).





**Supplementary Figure 6.** Anthocyanins in Syrah wines at pressing (A), post MLF (B), bottling (C), and accelerated aging (D) as malvidin-3-glucoside equivalents. Significant letters are provided, indicating statistical differences in anthocyanins between treatments at each time point using the Tukey-Kramer HSD test and p < 0.05.


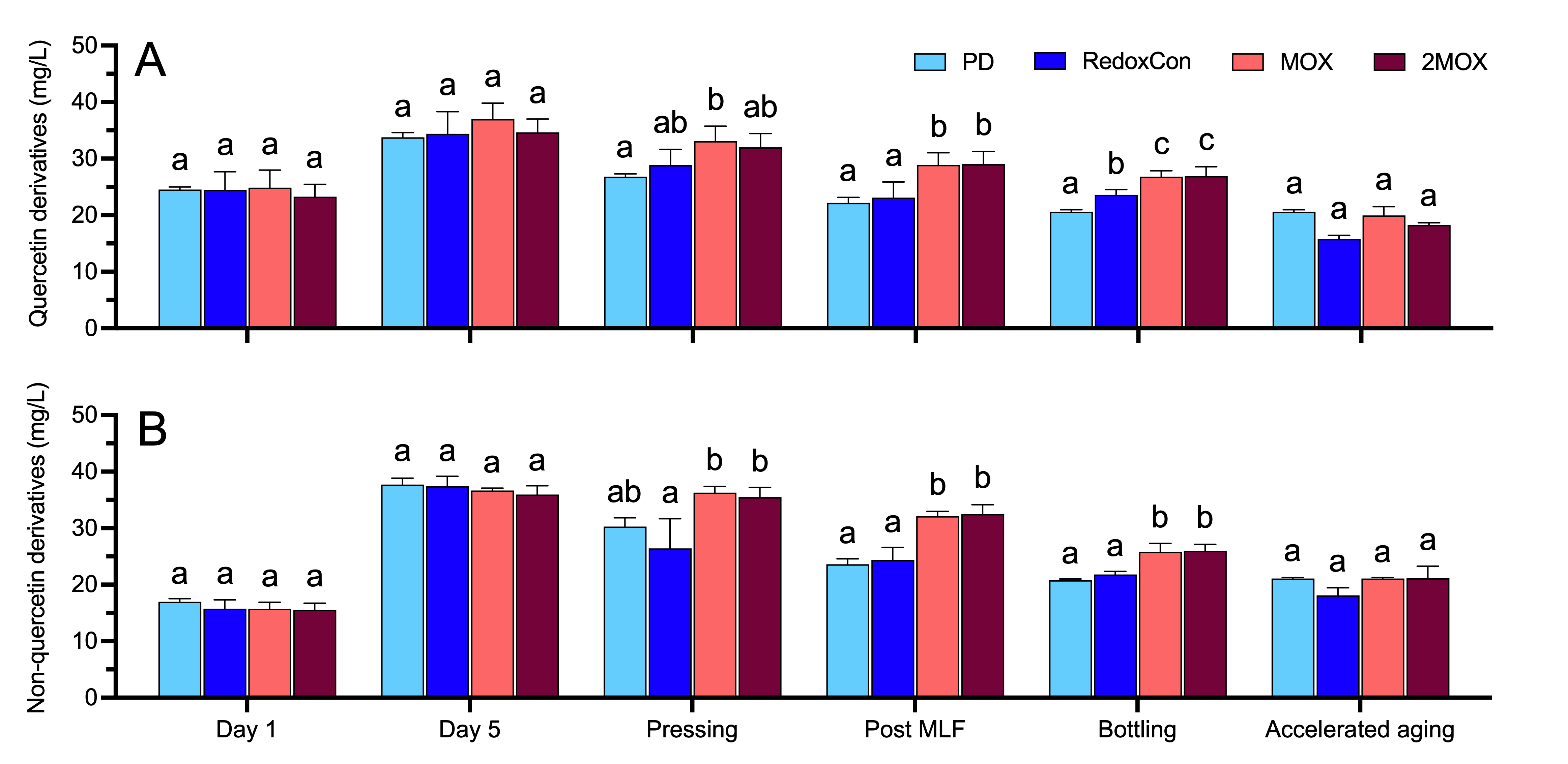


**Supplementary Figure 7.** Quercetin-derived (A) and non-quercetin-derived (B) flavonols in Syrah wines at day 1, day 5, pressing, post MLF, bottling, and accelerated aging. Significant letters are provided, indicating statistical differences in total quercetin and non-quercetin derived compounds between treatments at each time point using the Tukey-Kramer HSD test and p < 0.05.


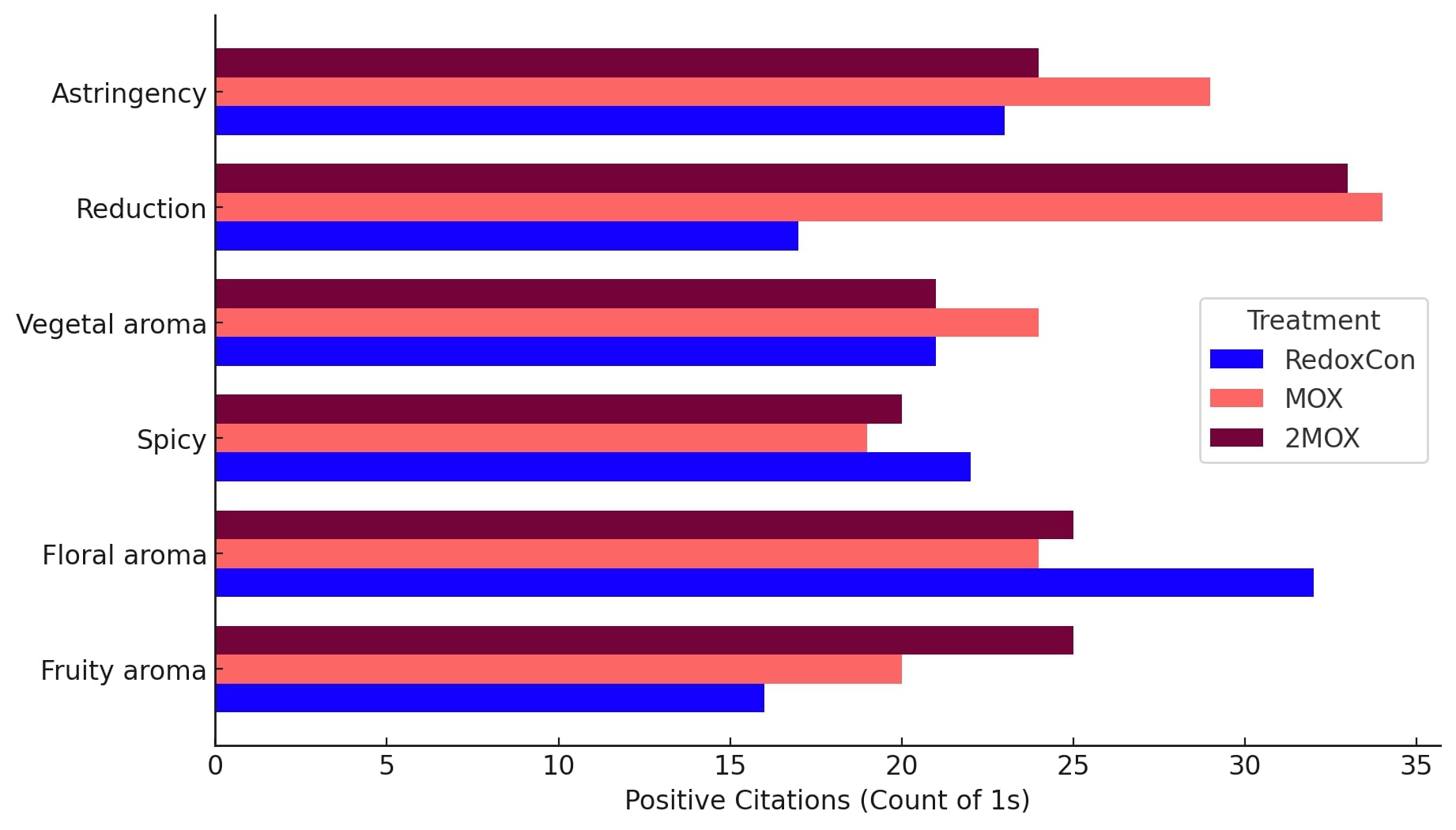


**Supplementary Figure 8.** Positive citation frequency plot of Syrah wines after sensory evaluation.
